# Identification of tRFs and phasiRNAs in tomato (*Solanum lycopersicum*) and their responses to exogenous abscisic acid

**DOI:** 10.1186/s12870-020-02528-8

**Published:** 2020-07-07

**Authors:** Wei Luan, Ya Dai, Xin-Yu Li, Yan Wang, Xiang Tao, Cai-Xia Li, Ping Mao, Xin-Rong Ma

**Affiliations:** 1 Chengdu Institute of Biology, Chinese Academy of Sciences, No.9, Section 4, Renmin South Road, Chengdu, 610041 Sichuan People’s Republic of China; 2 University of Chinese Academy of Sciences, Beijing, China

**Keywords:** tRF, phasiRNA, Abscisic acid (ABA), RNA-Seq, Transcription factors, Resistance genes

## Abstract

**Background:**

The non-coding small RNA tRFs (tRNA-derived fragments) and phasiRNAs (plant-specific) exert important roles in plant growth, development and stress resistances. However, whether the tRFs and phasiRNAs respond to the plant important stress hormone abscisic acid (ABA) remain enigma.

**Results:**

Here, the RNA-sequencing was implemented to decipher the landscape of tRFs and phasiRNAs in tomato (*Solanum lycopersicum*) leaves and their responses when foliar spraying exogenous ABA after 24 h. In total, 733 tRFs and 137 phasiRNAs were detected. The tRFs were mainly derived from the tRNA^Ala^ transporting alanine, which tended to be cleaved at the 5^**’**^terminal guanine site and D loop uracil site to produce tRF^Ala^ with length of 20 nt. Most of phasiRNAs originated from *NBS-LRR* resistance genes. Expression analysis revealed that 156 tRFs and 68 phasiRNAs expressed differentially, respectively. Generally, exogenous ABA mainly inhibited the expression of tRFs and phasiRNAs. Furthermore, integrating analysis of target gene prediction and transcriptome data presented that ABA significantly downregulated the abundance of phsaiRNAs associated with biological and abiotic resistances. Correspondingly, their target genes such as *AP2/ERF*, *WRKY* and *NBS-LRR*, *STK* and *RLK*, were mainly up-regulated.

**Conclusions:**

Combined with the previous analysis of ABA-response miRNAs, it was speculated that ABA can improve the plant resistances to various stresses by regulating the expression and interaction of small RNAs (such as miRNAs, tRFs, phasiRNAs) and their target genes. This study enriches the plant tRFs and phasiRNAs, providing a vital basis for further investigating ABA response-tRFs and phasiRNAs and their functions in biotic and abiotic stresses.

## Background

RNA is a single strand of nucleotide formed by transcription using a strand of DNA as a template and following the principle of complementary base pairing. For a long time, it has been widely recognized that RNA functions only as an intermediate to carry genetic information from DNA to protein. However, studies over the past decade have shown that RNA plays important roles in the process of life than previously understood.

Plant endogenous small RNA (sRNA) is generally between 20 and 24 nt in length, which is initially produced as double-stranded duplexes formed the helical region of the larger RNA precursor by the endonucleases activities of Dicer-like (DCL) protein [7]. By binding with Argonaute (AGO) protein and targeting target RNA, it exerts a negative post-transcriptional regulation function and plays an important role in plant growth and development, stress response and epigenetic modification [20].

tRNA is a class of widely existed and abundant non-coding RNA that is generally between 72 and 90 nt in length. In addition to participating in protein synthesis, tRNA also has a variety of non-canonical functions, such as biological processes involved in cell proliferation, differentiation, apoptosis, and stress response [42, 46]. tRF, tRNA-derived fragment, is a class of non-coding sRNA, formed tRNA precursors or mature bodies by specific endonucleases activities under precise regulation. According to the different restriction sites, it is divided into five major types: 5e-tRF, D-tRF, A-tRF, V-tRF and 3e-tRF [44]. tRF, first found in HeLa cells [33], widely exists in eukaryotes and prokaryotes, and has a variety of biological functions, including as a gene expression regulator and as a signaling molecule in stress response [70]. At present, the research on tRF is mainly carried out on animals. A little studies on plant tRF have been reported in *Arabidopsis thaliana* [4, 24, 37], *Oryza sativa* [4, 41], *Physcomitrella patens* [4, 41], *Cucurbita maxima* [66], *Brassica rapa* [10] and *Triticum aestivum* [59]. The research by Alves et al. [4] shown that the production mechanism of tRF in *Arabidopsis thaliana* is different from that of miRNA, not depending on DCL proteins, but the specific mechanism is not yet clear, it is speculated that S-like Ribonuclease 1 (RNS1) may be involved in the formation of tRF; co-immunoprecipitation experiments showed that both animal and plant tRF could participate in RNAi in combination with AGO protein family, and could also regulate gene expression and protein synthesis through interaction with other sRNA. Furthermore, Alves et al. also revealed the expression patterns of tRF in *Arabidopsis thaliana*, *Oryza sativa* and *Physcomitrella patens* under drought, cold and oxidative stress [4]. Reportedly, tRF could inhibit transposable elements and maintain genomic stability by complementing the target gene in *Arabidopsis thaliana* and rice pollen [35, 41]. However, the method to predict target genes of tRF is still not accurate.

Phased, secondary, small interfering RNA (phasiRNA) is a class of plant-specific non-coding siRNA with phase alignment structure, which is mediated by miRNAs at protein coding sites and non-coding sites (collectively referred to as phasiRNA producing loci, *PHAS* loci). Depending on its mode of action, phasiRNA can be divided into cis-acting siRNA (casiRNA) and trans-acting siRNA (tasiRNA) [5, 13, 65]. The primary transcript of the *PHAS* gene transcribed by RNA polymerase II binds to the polyribosome on the rough endoplasmic reticulum and is cleaved by the miRNA-mediated AGO protein to generate a phasiRNA precursor; then it is replicated into double-stranded RNA by RDR6 (RNA-dependent RNA polymerase 6) assisted with SGS3 (suppressor of gene silencing 3) and SDE5 (silencing defective5); double-stranded RNA is cleaved by DRB4 (dsRNA-binding factor 4) and DCL4 (Dicer like 4) complexes into head-to-tail short RNA fragments with length of 21 nt or 24 nt, i.e. phasiRNA [69]. PhsiRNA can participate in plant reproductive regulation by regulating cell division and differentiation, affect the development of tissues and organs during plant growth and development [8, 19]. In addition, phasiRNA also play important role in resisting abiotic stress [34, 51, 54] and pathogen infection [26, 61, 68]. For example, the phasiRNAs produced in miR6445-*PHA18* pathway and miR393-*PHA1/2* pathway can regulate the response of *Populus trichocarpa* to drought and salt stress respectively [51]. In addition, the trans-acting of phasiRNA enhances tomato resistance to potato spindle tuber viroid (PSTVd) [68].

Abscisic acid can be divided into S-ABA and R-ABA according to its optical configuration, and active natural abscisic acid is S-ABA (for short ABA). As important endogenous hormone in plants, ABA can promote the abscission of flowers, leaves and fruits and the maturation and dormancy of seeds, regulate stomatal movement and blossom, inhibit growth and accelerate senescence in the process of plant growth and development [2, 49, 52, 62]. Moreover, ABA can improve the tolerance of plants under drought, temperature, high salt, heavy metals and other stresses, so it is also called stress hormone [29, 32, 38, 43, 71]. Different studies on ABA in plants response to pathogens have contrasting conclusions [39]. Whether ABA plays a positive or negative regulation in plant disease resistance depends on the type of pathogen, the mode of infection, the defense period, the type of tissue affected and the concentration of ABA [28]. Our previous studies showed that low concentration of ABA could significantly improve the resistance of tomatoes to multiple pathogens, inhibit the production of miRNAs targeting disease-resistant genes, and promote the expression of disease-resistant genes [14, 60], which was consistent with our field experiments.

Tomato (*Solanum lycopersicum*) (2n = 24) is an important horticultural economic crop with rich genetic resources, multiple mutants, small genome (950 Mb), extensive genetic map, efficient and stable genetic transformation system, easy hybridization and high reproductive coefficient. With the completion of tomato genome sequencing in 2012, it has become an important model plant for crop research [15].

At present, there are few studies on plant tRFs and phasiRNAs, and their responses to ABA remain an enigma. In this study, tRFs and phasiRNAs of tomato responding to exogenous ABA were identified and analyzed by small RNA sequencing. And the correlation between differentially expressed phasiRNAs and their target genes expressions was analyzed, inferring a consequence that ABA boosts plant disease resistance and stress adaption. The results not only enriched plant tRFs and phasiRNAs, but also provided an important basis for further research on the role of tRFs and phasiRNAs in plants responding to ABA and adaptation to biotic and abiotic stresses.

## Methods

### Plant materials

The seeds of tomato cv. Hongtaiyang 903 (a cultivar that has been commercially promoted) were bought from Dalian Tiandi Seed CO.. LTD. Plants were cultivated and treated by ABA solution and deionized water as previously described [60]. Briefly, seeds of tomato were sowed in plastic pots and grown in a greenhouse under natural light in Chengdu, Sichuan Province, China. When the tomatoes grew 5–7 leaves, they were randomly divided into two groups. One group was sprayed with 2 mg/L ABA aqueous solution as the treatment group (A1D); the other group was sprayed with the same volume of deionized water as the control group (C1D). Then the third leaf was collected and combined as a mixed sample of ten plants separately from the two groups after 24 h, snap-frozen and stored immediately in liquid nitrogen for subsequent experiments.

### sRNA sequencing and library construction

The materials of tomato transcriptome sequencing and sRNA sequencing were same, and the sequencing was completed in 2013 and 2016, respectively [14, 60]. Total RNA was isolated from C1D and A1D tomato leaves using Trizol reagent (Invitrogen, Carlsbad, CA). Small RNAs in length of 18–30 nt were separated from total RNA by a 15% PAGE gel, then were purified from the gel. Then small RNAs were reversely transcribed into cDNAs through reverse transcription PCR. The cDNAs were sequenced on Illumina HiSeq™ 2000 by BGI (Shenzhen, China). The raw reads obtained from sequencing were optimized and compared to tomato genome for sRNA annotation and analysis of expression and distribution in the genome.

### Identification and quantification of tRF and phasiRNA

For tRFs identification, the tRNAscan-se software (http://lowelab.ucsc.edu/tRNAscan-SE/) was used to obtain the secondary structure information of the tomato tRNA sequence, and to identify each “leaf” sequence of tRNA “clover”. According to the classification principle of Olvedy [44], the secondary structure of tRNA was divided into five parts including 5e-tRF, D-tRF, A-tRF, V-tRF and 3e-tRF. The short reads obtained from sequencing were aligned to the tRNA using the bowtie software (http://bowtie-bio.sourceforge.net/index.shtml), and the alignment mode was no mismatching. According to the comparison results, the short reads peak on tRNA identified by flaimapper software [64] was regarded as a tRF. Calculating the proportion of the intersection between a tRF and a certain part of the secondary structure of tRNA, if the proportion reaches 75%, the tRF is classified into the part. Because of the long intersection of some parts of tRNA, if there is a tRF falling into the intersection, the tRF will be simultaneously classified into two categories. If a tRF cannot occupy an intersection of 75% of the total length in any part, the tRF is classified as others. Then, tRF was quantified based on the reads comparison of the tRNA of each sample. For each read, if three or fewer bases are located outside a certain tRF, it is determined that the read is from this tRF, and the number of repetitions of the read is the expression level or abundance of this tRF.

According to the method of Guo et al. [22], use phaseTank (http://phasetank.sourceforge.net/) to compare all short reads to the reference genome, identify candidate phasiRNAs and their precursors. PhaseTank uses a new scoring system to predict *PHAS* loci and phasiRNA regulatory networks on a genome-wide scale. The phased score of a sequence is determined by phased ratio, number and abundance. The larger the score, the greater the possibility that the sequence is a true *PHAS* gene. Then, the candidate phasiRNA precursor sequences intersect with the known gene element sequences in the reference genome, excluding the false positive results such as tRNA and rRNA, and the remaining long fragments are phasiRNA precursors. The short sequence of 21 nt in length from these precursors is phasiRNA, and the number of repetitions of phasiRNA appearing in the corresponding position of the genome is the expression level of phasiRNA in each sample.

### Differential expression analysis of tRF and phasiRNA

The reads of tRF and phasiRNA and reads of miRNA [14] were normalized as a whole, and the unit of normalized expression data was reads per million (RPM).

Normalized counts of a tRF = (the reads number of a tRF/the reads number of tRFs, phasiRNAs and miRNAs)*1,000,000.

Normalized counts of a phasiRNA = (the reads number of a phasiRNA/the reads number of tRFs, phasiRNAs and miRNAs)*1,000,000.

After normalizing the expression of tRF and phasiRNA in the two samples, differential expression of tRF and phasiRNA were analyzed separately. The fold-change was calculated by dividing the tRF RPM in A1D by the tRF RPM in C1D, the same as phasiRNA. *P*-value was calculated as described by Audic [6]. Changes in the expression level of at least |log_2_fold-change (log_2_FC)| ≥0.25 and *P*-value < 0.05 were recognized as a response to ABA treatment, and significant difference expression of tRF or phasiRNA was assigned to sequences with |log_2_FC| ≥1. tRF and phasiRNA with |log_2_FC| < 0.25 were considered to have no obvious change in expression levels.

### phasiRNA target prediction

Because the method of tRF target prediction is still immature, only phasiRNA target genes have been predicted in this study referring to the method of Enright et al. [18]. The method of phasiRNA target prediction based on three properties [1, 36]: the sequence complementarity score calculated using the miRanda algorithm, the free energy of the RNA-RNA duplexs and the conservation of target sites in the related genomes. In generally, if the score is higher and the free energy is lower, then the targeting relationship is stronger. Here, the threshold set in this study is: score ≥ 500 and free energy ≤ − 50.

## Results

### Sequencing results of sRNA

We have constructed sRNA libraries from tomato leaves of the ABA treatment group (A1D) and control group (C1D) respectively, which covers almost all sRNA, including miRNAs, siRNA, tRNA, rRNA, snRNA, snoRNA, exon or intron degradation fragments [14]. By annotating these sRNA clean reads into the tomato genome, 733 tRFs (545 in C1D and 536 in AID) and 137 phasiRNAs were identified. Furthermore, it was found that miRNAs accounted for a higher proportion in the annotated sRNAs of both C1D and A1D. Therefore, we carried out an overall normalization analysis of the expression levels of tomato tRFs, phasiRNAs and miRNAs to explore the proportion of tRFs and phasiRNAs in these sRNAs and their responses to exogenous ABA.

**Fig. 1:**
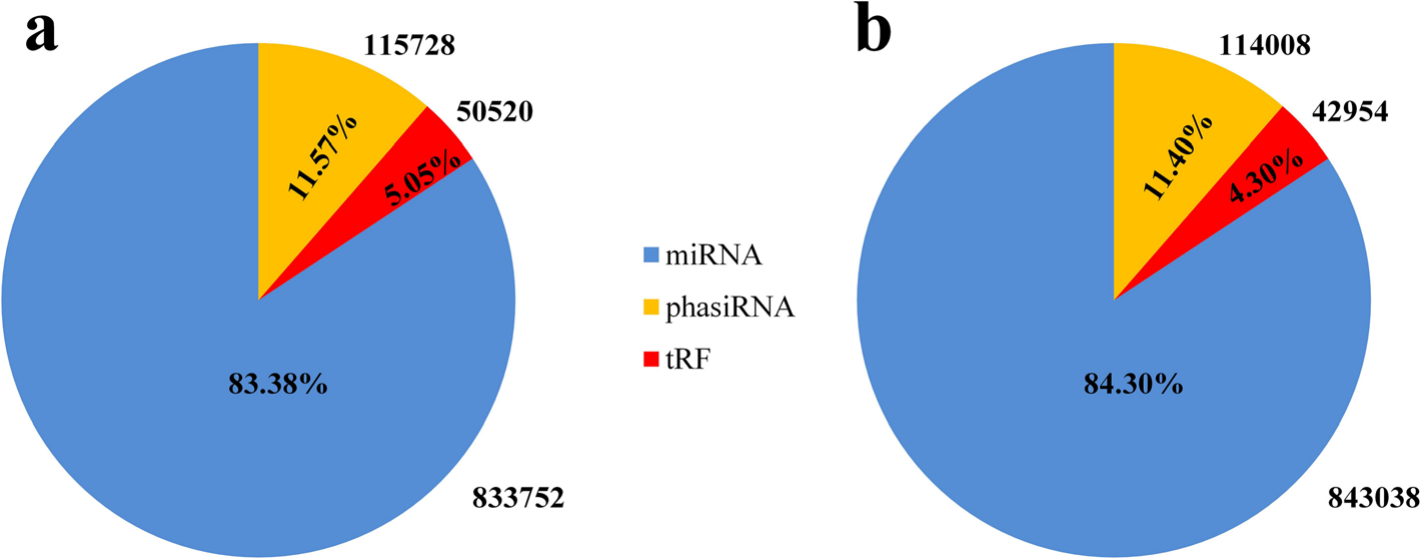
The overall expression levels of miRNAs, tRFs and phasiRNAs in C1D (**a**) and A1D (**b**)

The overall normalization analysis of the expression of the three kinds of sRNAs, tRFs, phasiRNAs and miRNAs showed that in the control group (C1D), the total expression of miRNAs was 833,750 RPM (accounting for 83.38%) while the total expression of tRFs was 50,520 RPM (5.05%) and that of phasiRNAs was 115,728 RPM (11.57%) (Fig. 1a). Similarly, the total expression of each sort of sRNA in the treatment group (A1D) was 843,038 RPM (accounting for 84.30%) for miRNAs, 42,954 RPM (4.30%) for tRFs, and 114,008 RPM (11.40%) for phasiRNAs (Fig. 1b). In addition, compared with C1D, the expression of miRNAs, tRFs and phasiRNAs in A1D decreased, with a total reduction of 9288, 9566 and 1720 RPM, respectively.

### Response of tomato tRFs to exogenous ABA and the differential expression analysis

#### The features of tRFs

**Fig. 2:**
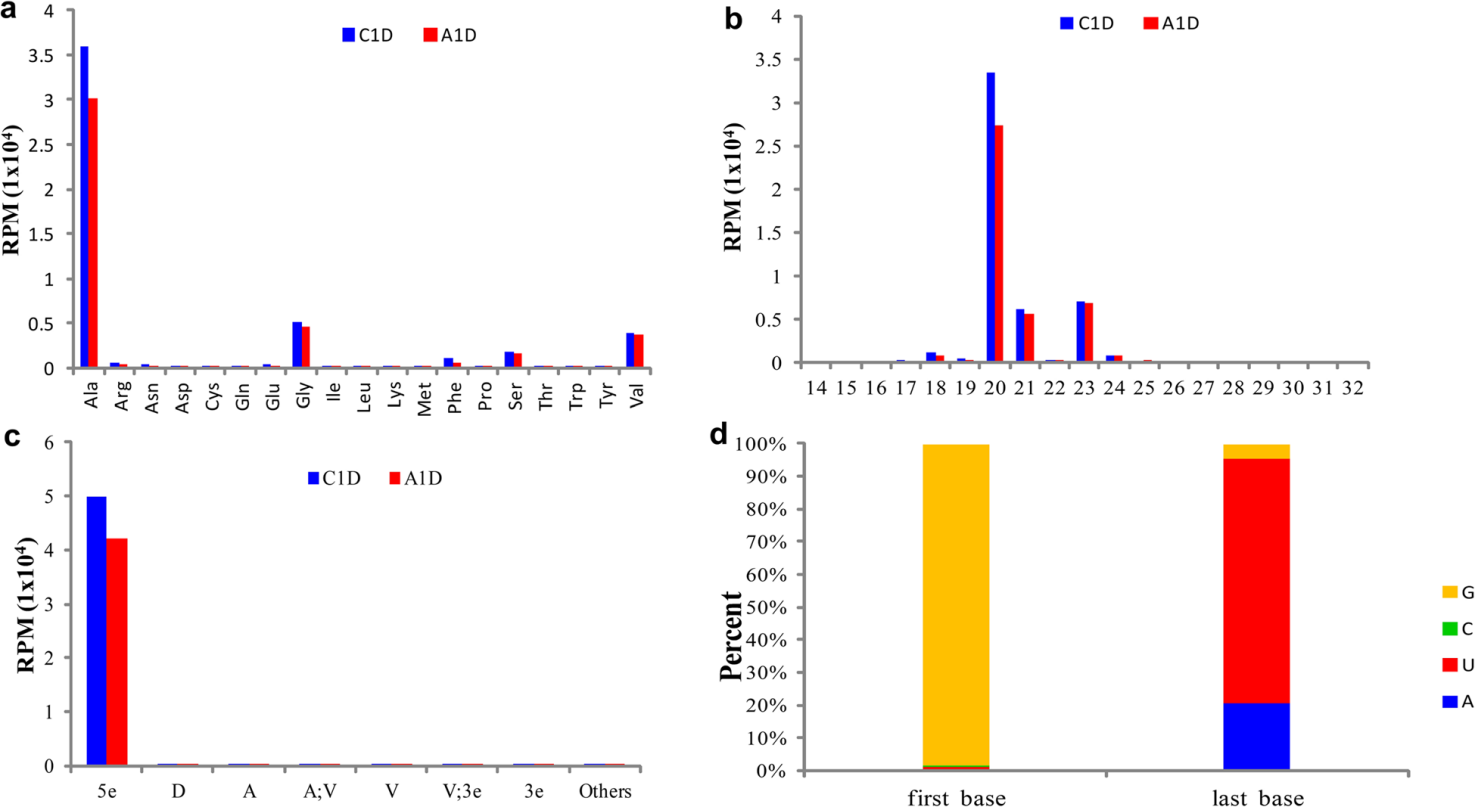
The origin of tRFs and their total abundances (**a**), length and abundances (**b**), type and abundances (**c**), and preference of 5e-tRF first and last base in C1D (**d**)

We analyzed the origin, length, type and base preference of tRF identified in C1D and A1D, respectively. The results were basically consistent but different (Fig. 2). The identified tRFs originated from tRNAs except the kind of tRNA^His^ (tRNA transporting histidine, and so on below), most of which originated from tRNA^Ala^, followed by tRNA^Gly^, tRNA^Val^ and tRNA^Ser^ (Fig. 2a).

The length analysis showed that the tRFs was mainly 20 nt in length (Fig. 2b). And mostly of the tRFs were included in the range of 5e-tRF with an absolute dominant proportion of 98.67% in C1D and 98.35% in A1D, respectively (Fig. 2c). Analyzing the preference of the first and last bases of these 5e-tRFs in C1D and A1D, it was found that the majority of 5^**’**^ terminal bases were guanine (G), separately accounted for 98.57 and 98.93%, while the 3^**’**^ was uracil (U) accounting for 74.86 and 73.79% respectively (Fig. 2d). Because the base preference of 5e-tRF in A1D and C1D is basically the same, Fig. 2 shows only the base distribution of 5e-tRF in C1D.

In summary, the identified tRFs in C1D and A1D originated mainly from tRNA^Ala^, and tended to produce tRF^Ala^ (tRFs originating from tRNA^Ala^, and so on below) with length of 20 nt by cutting at tRNA 5^**’**^ terminal guanine site and D loop uracil site (the sort of 5e-tRF).

#### Differential expression analysis of tRF

In general, compared with C1D, the total expression of tRFs in A1D was obviously decreased (from 50,520 to 42,954 RPM), especially tRF^Ala^. And only 5 kinds of tRFs increased slightly, namely tRF^Asp^, tRF^Cys^, tRF^Pro^, tRF^Thr^ and tRF^Trp^ (Fig. 2A). Classified by length, the expression of tRFs with length of 15–23 nt decreased in A1D compared with C1D. In particular, the tRFs with a length of 20 nt had the highest abundance and the largest decrease at 6081.12 RPM (Fig. 2b).

**Fig. 3:**
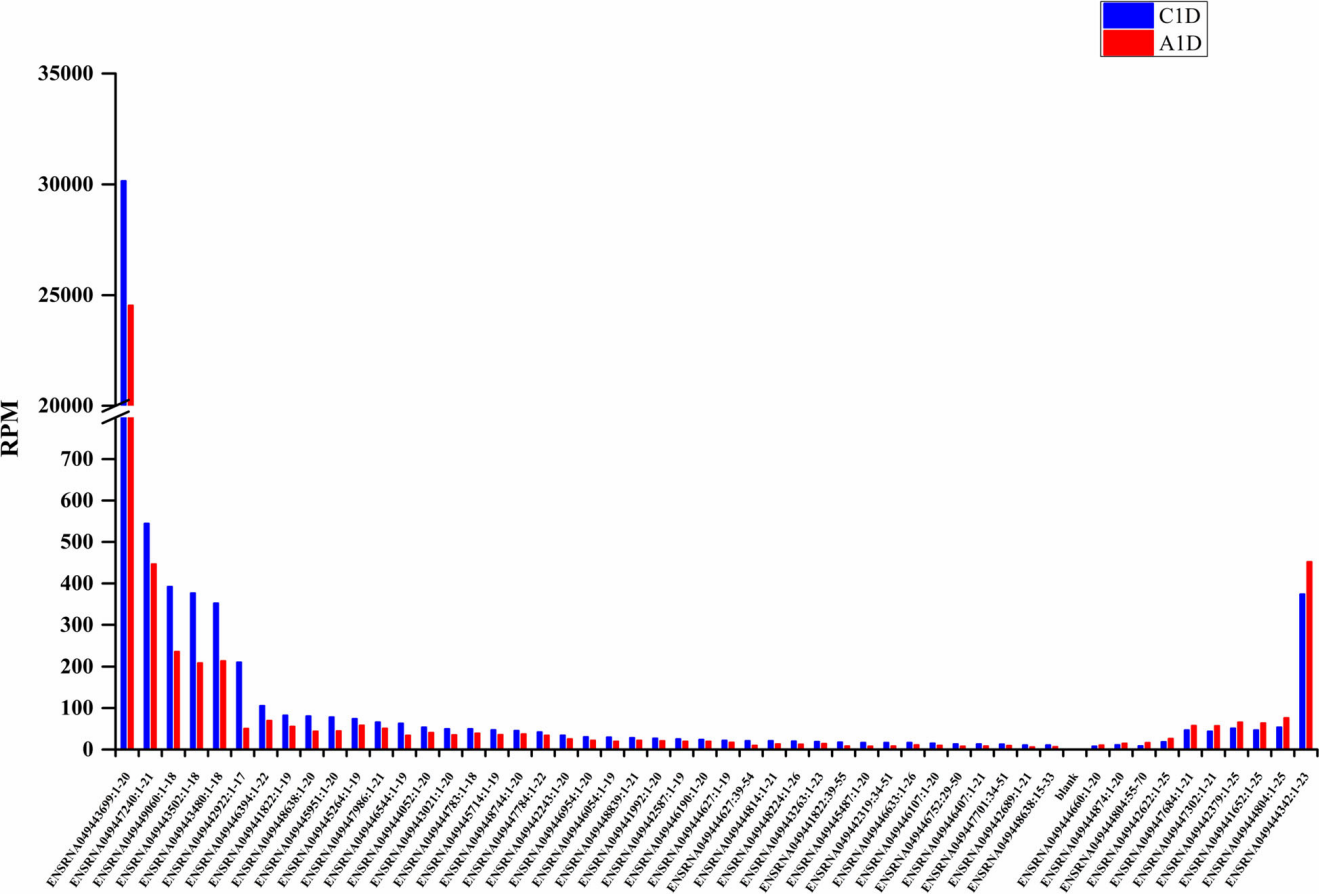
Differentially expressed tRFs that have a minimum RPM value of ten in one library are listed in A1D and C1D

A total of 733 tRFs were identified in C1D and A1D (Supplementary Table S1). In order to reduce noise, the tRFs whose expression level is lower than 2 RPM in both C1D and A1D were removed, then 230 tRFs were left for subsequent differential expression analysis. Among the 230 tRFs, 156 were differentially expressed (|log_2_FC| ≥ 0.25 and *p* < 0.05), of which 74 were up-regulated and 82 were down-regulated; and 74 had no significant change (|log_2_FC| < 0.25) (Supplementary Table S2). Of the 156 differentially expressed tRFs, 51 expressed more than 10 RPMs in at least one library (Supplementary Table S3). For the 51 tRFs, their expression levels were taken as ordinates to observe the changes in C1D and A1D (Fig. 3). The results showed that 41 of the 51 tRFs were down-regulated, and the total abundance of down-regulated tRFs decreased from 33,315.52 to 26,572.77 RPM, with a decrease of 6742.75 RPM; while the up-regulated tRFs increased from 663.41 to 840.83 RPM, only with an increase of 177.42 RPM. The tRF (ID: ENSRNA049443699:1–20), one kind of tRF^Ala^, expressed 30,155.81 RPM in C1D and 24,539.08 RPM in A1D with decline of 5616.73 RPM, which was much higher than that of other tRFs (less than 600 RPM). The results indicated that spraying exogenous ABA generally inhibited the expression of tRFs, especially the 20 nt tRF^Ala^. In addition, the expression of tRF^Asp^ increased from 166.1 to 236.72 RPM, and the expression of tRF^Glu^ decreased from 491.46 to 326.82 RPM.

### Response of tomato phasiRNAs to exogenous ABA and the correlation with stress resistance

#### *PHAS* loci

A total of 137 phasiRNAs were identified in C1D and A1D (Supplementary Table S4). Analysis of *PHAS* loci revealed that 110 phasiRNAs originated from protein-coding genes and 27 phasiRNAs originated from non-coding genes. Of the 110 phasiRNAs originating from protein coding genes, 60 originated from *NBS-LRR* (nucleotide binding site- Leucine rich repeat) gene, 4 originated from *RLK* (receptor-like kinase) gene, 1 originated from disease resistance protein R3a-like coding gene, 7 originated from protein coding genes encoding Zinc finger CCCH domain, 2 originated from auxin F-box protein 5 coding genes, 2 originated from putative Os03g0370250 protein coding gene, and 34 originated from unannotated protein coding genes (Table 1).

**Table 1:** *PHAS* loci of tomato

*PHAS* loci	number
*NBS-LRR*	60
*RLK*	4
Disease resistance protein R3a-like protein	1
Zinc finger CCCH domain-containing protein 34	4
Zinc finger CCCH domain-containing protein 58	3
Auxin F-box protein 5	2
Putative Os03g0370250 protein	2
Unannotated protein	34
Noncoding	27

#### Differential expression analysis of phasiRNA

**Fig. 4:**
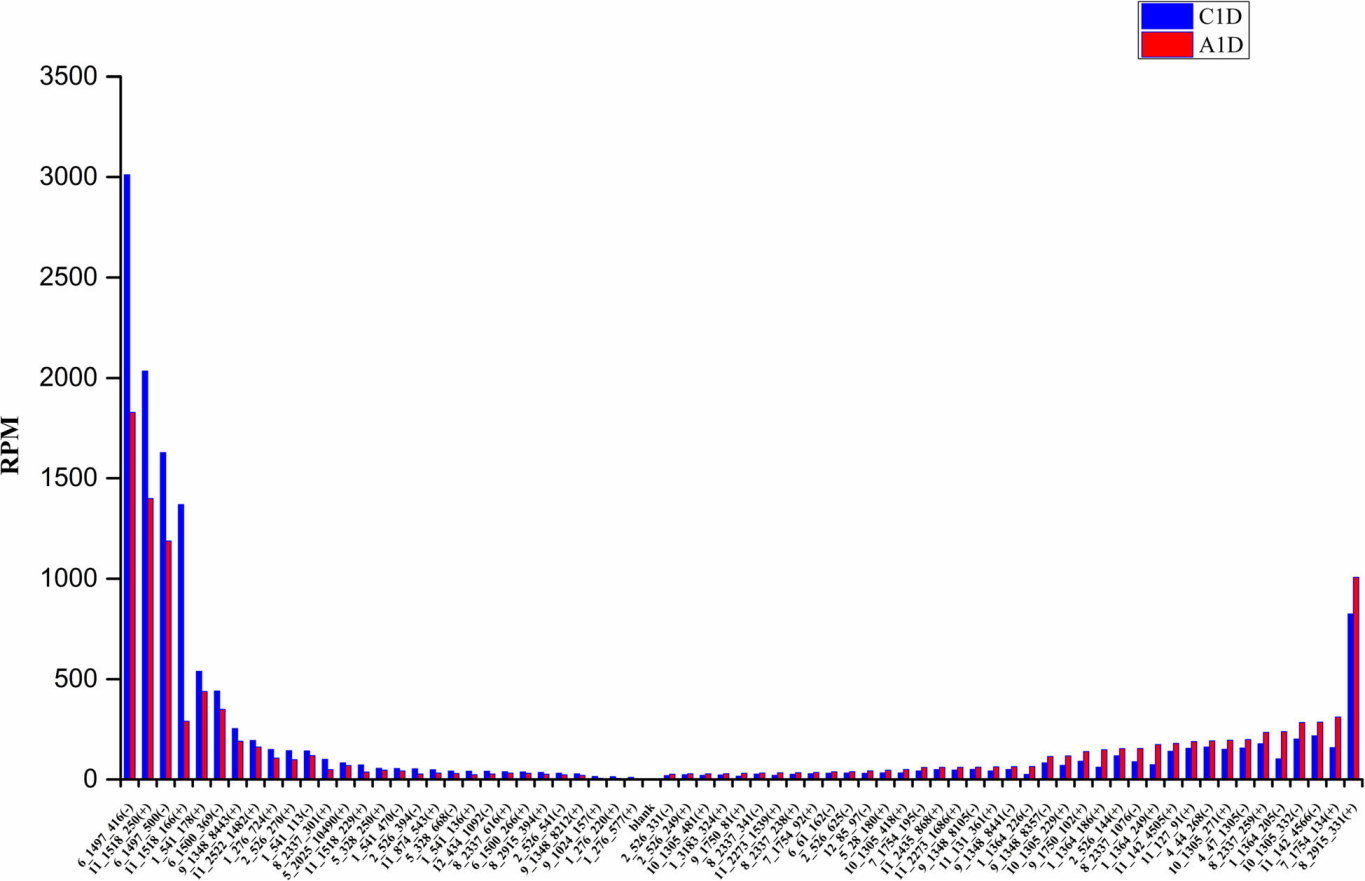
Differentially expressed phasiRNAs in A1D and C1D

The all 137 phasiRNAs had a minimum RPM value of ten in at least one library, thus can be directly used for differential expression analysis (Supplementary Table S5). Among them, 68 were differentially expressed (|log_2_FC| ≥ 0.25 and *p* < 0.05), of which 39 were up-regulated and 29 were down-regulated; and 69 had no significant change (|log_2_FC| < 0.25) (Supplementary Table S6). Among the 68 phasiRNAs, although the down-regulated phasiRNAs (29) were less than the up-regulated ones (39), the abundance and decline of down-regulated phasiRNAs were much higher than that of the up-regulated phasiRNAs (Fig. 4). The total abundance of the down-regulated phasiRNAs reduced from 10,715.98 to 6710.06 RPM with a decrease of 4005.92 RPM, and that of the up-regulated phasiRNAs increased from 3715.64 to 5529.62 RPM with only an increase of 1543.98 RPM. The results showed that the phasiRNAs respond to ABA were higher abundance and ABA generally inhibited their expressions.

#### Differentially expressed phasiRNAs targeting stress-related genes

In addition, using the method of Enright et al. [18] to predict the target genes of the 68 differentially expressed phasiRNAs, and the resistance-related phasiRNAs were known accordingly (Supplementary Table S7). Furthermore, the comparative transcriptome data of ABA responses were combined to analyze the effect of abundance changes of these phasiRNAs on target gene expressions (Supplementary Table S8).

The results showed that 15 differentially expressed phasiRNAs were predicted to target resistance-related genes, including 7 phasiRNAs target TFs and 12 target resistance genes (R genes). The TFs include AP2/ERF (APETALA 2 / ethylene-responsive element binding factor), MYB (v-myb avian myeloblastosis viral oncogene homolog), NAC, ARF (Auxin response factor), GRAS, bHLH (basic helix-loop-helix protein) and WRKY. And the R genes include *NBS-LRR*, *STK* (serine/threonine-protein kinase) and *RLK* (Table 2).

**Table 2:** Effect of exogenous ABA on stress-related phasiRNA and target gene expression

phasiRNA ID	C1D (RPM)	A1D (RPM)	Target genes (No.)	Expressed transcripts & the numbers			
				Sum	up-regulated	down-regulated	no changed
**1_541_136(+)**	**41.03**	**24.16**	**NBS-LRR(2)**	**2**	**1**	**0**	**1**
			^**d**^**STK(2)**	**6**	**3**	**2**	**1**
**1_541_178(+)**	**539.81**	**439.36**	**RLK(1)**	**1**	**0**	**0**	**1**
**6_1497_500(−)**	**1628.21**	**1188.46**	**NBS-LRR(1)**	**4**	**1**	**0**	**3**
			**STK(1)**	**1**	**1**	**0**	**0**
			**RLK(3)**	**7**	**5**	**1**	**1**
**6_1500_369(−)**	**441.61**	**350.39**	**RLK(1)**	**1**	**0**	**0**	**1**
**11_1518_166(+)**	**1368.32**	**291.07**	**AP2/ERF(1)**	**1**	**1**	**0**	**0**
			**bHLH(1)**	**2**	**1**	**0**	**1**
			**GRAS(1)**	**2**	**0**	**0**	**2**
			**WRKY(2)**	**3**	**3**	**0**	**0**
			^**D**^**STK(7)**	**12**	**5**	**3**	**4**
			^**E**^**RLK(16)**	**27**	**12**	**2**	**13**
**11_1518_229(+)**	**72.79**	**37.35**	**AP2/ERF(3)**	**2**	**2**	**0**	**0**
			**ERF(1)**	**–**			
			**MYB(3)**	**5**	**1**	**0**	**4**
			**NAC(2)**	**1**	**0**	**0**	**1**
			**NBS-LRR(1)**	**1**	**1**	**0**	**0**
			**STK(3)**	**4**	**3**	**0**	**1**
			**RLK(1)**	**2**	**1**	**1**	**0**
**11_1518_250(+)**	**2035.14**	**1398.8**	**ARF(1)**	**1**	**1**	**0**	**0**
**1_1364_205(−)**	**103.08**	**239.45**	^**a**^**ARF(1)**	**1**	**0**	**0**	**1**
			^**b**^**STK(3)**	**4**	**2**	**0**	**2**
**1_1364_226(−)**	**25.4**	**64.8**	^**a**^**ARF(1)**	**1**	**0**	**0**	**1**
			^**b**^**STK(3)**	**4**	**2**	**0**	**2**
**1_1364_249(+)**	**74.25**	**174.64**	^**c**^**MYB(1)**	**–**			
**9_1750_102(+)**	**91.35**	**139.5**	**NBS-LRR(1)**	**2**	**1**	**1**	**0**
			**RLK(1)**	**1**	**0**	**0**	**1**
**10_1305_229(+)**	**70.83**	**118.08**	^**c**^**MYB(1)**	**–**			
**10_1305_418(+)**	**32.73**	**49.98**	^**d**^**STK(1)**	**2**	**1**	**1**	**0**
**11_131_361(+)**	**42.99**	**63.16**	**NBS-LRR(6)**	**9**	**4**	**2**	**3**
			^**d**^**STK(4)**	**9**	**4**	**2**	**3**
			^**e**^**RLK(2)**	**2**	**0**	**0**	**2**
**11_2273_1539(+)**	**20.52**	**33.5**	**NBS-LRR(2)**	**3**	**1**	**1**	**1**
			**RLK(1)**	**1**	**1**	**0**	**0**

Note: the same letters ^a^, ^b^ and ^c^ denote the same target gene; ^d^ these STK genes are included in the ^D^7 STKs targeted by 11_1518_166(+); ^e^ these RLK genes are included in the ^E^16 RLKs targeted by 11_1518_166(+)

Overall, although the resistance-related phasiRNAs were down-regulated (7) less than the up-regulated (8), the abundance and extent of variation of the down-regulated phasiRNAs were much higher than those of the up-regulated phasiRNAs. Correspondingly, most resistant target gene expressions were up-regulated. Moreover, some phasiRNAs target multiple genes, and similarly, a gene can be targeted by multiple phasiRNAs.

In the 7 differentially expressed phasiRNAs that targeted TFs, 4 up-regulated and 3 down-regulated. Their expressions and the influence on target genes were in line with the above-mentioned results. In particular, some down-regulated phasiRNAs target multiple genes with large decline, such as 11_1518_166(+) (1368.32 to 291.07 RPM) and 11_1518_229(+) (72.79 to 37.35 RPM). The two phasiRNAs targeted five *AP2/ERF* genes (including an *ERF* gene), and three transcripts were detected and elevated in transcript data (Table 2).

Four *MYB* genes were targeted by three phasiRNAs containing 1_1364_249(+), 10_1305_229(+) and 11_1518_229(+). Up-regulated 1_1364_249(+) (74.25 to 174.64 RPM) and 10_1305_229(+) (70.83 to 118.08 RPM) targeted a same *MYB* gene, but no transcripts were detected. The down-regulated 11_1518_229(+) also targeted five transcripts of three *MYB* genes, of which one transcript was elevated and four transcripts were unaltered.

In addition, the down-regulated 11_1518_229(+) also targeted two *NAC* genes, but detected only one transcript with no alteration. Besides, two *ARF* genes was targeted by three phasiRNAs, namely 1_1364_205(−), 1_1364_226(−) and 11_1518_250(+). Of which, the up-regulated 1_1364_205 (−) (103.08 to 239.45 RPM) and 1_1364_226 (−) (25.4 to 64.8 RPM) targeted a same *ARF* gene, and one detected transcript was unaltered. However, the down-regulated 11_1518_250(+) (2035.14 to 1398.8 RPM) targeted one transcript of another *ARF* gene, which was elevated.

*GRAS*, *bHLH* and *WRKY* were predicted to be simultaneously targeted by the down-regulated 11_1518_166(+). It was predicted to target two transcripts of one *GRAS* gene, which were unaltered. It was also predicted to target two transcripts of one *bHLH* gene, of which one transcript was elevated and the other one was unaltered. In addition, it was predicted to target three transcripts of two *WRKY* genes, all of which were elevated.

In short, a total of 7 differentially expressed phasiRNAs are predicted to target the above TFs. Three down-regulated phasiRNAs are predicted to target a total of 17 transcripts of 15 TF genes, with nine that were increased in expression and eight remained unchanged. Four up-regulated phasiRNAs are predicted to target two TF genes, which were unaltered.

In this study, 12 differentially expressed phasiRNAs (6 up-regulated and 6 down-regulated) were predicted to target *NBS-LRRs*, *STK*s or *RLK*s. Combining transcriptome data to analyze the expression of target genes, the results showed that most of these ABA responsed target resistance genes were up-regulated.

Thirteen *NBS-LRR* genes (21 transcripts) were targeted by six phasiRNAs, including down-regulated 1_541_136(+), 6_1497_500(−) and 11_1518_229(+), and up-regulated 9_1750_102(+), 11_131_361(+) and 11_2273_1539(+). Among the 7 detected transcripts of 4 *NBS-LRR* genes, which targeted by the down expressed phasiRNAs, 3 were elevated and 4 changed unobviously. And of which the 14 detected transcripts of 9 *NBS-LRR* genes, which targeted by the up expressed phasiRNAs, 6 were elevated, 4 decreased, and 4 changed unobviously.

Fourteen *STK* genes (30 transcripts) were targeted by eight phasiRNAs, including down-regulated 1_541_136(+), 6_1497_500(−), 11_1518_166(+) and 11_1518_229(+), and up-regulated 1_1364_205(−), 1_1364_226(−), 10_1305_418(+) and 11_131_361(+). Among the 17 detected transcripts of 11 *STK* genes, which targeted by the down expressed phasiRNAs, 9 were elevated, 3 decreased and 5 changed unobviously. And of which the 13 detected transcripts of 7 *STK* genes, which targeted by the up expressed phasiRNAs, 6 were elevated, 2 decreased, and 5 changed unobviously. Besides, it was found that Solyc02g083900.2.1 was targeted by 1_541_136(+) and 11_131_361(+), and Solyc01g106770.2.1 was targeted by 1_541_136(+), 11_1518_166(+), and 11_131_361(+) simultaneously.

Twenty-four *RLK* genes (40 transcripts) were target by eight phasiRNAs, including down-regulated 1_541_178(+), 6_1497_500(−), 6_1500_369(−), 11_1518_166(+), 11_1518_229(+), and up-regulated 9_1750_102(+), 11_131_361(+), 11_2273_1539(+). Among the 38 detected transcripts of 22 *RLK* genes, which targeted by the down expressed phasiRNAs, 18 were elevated, 4 decreased and 16 changed unobviously. And of which the 4 detected transcripts of 4 *RLK* genes, which targeted by the up expressed phasiRNAs, 1 was elevated and 3 changed unobviously. Besides, it was found that Solyc12g056730.1.1 was targeted by 11_131_361(+) and 11_1518_166(+) simultaneously, corresponding to two transcripts.

In summary, there were 12 differentially expressed phasiRNAs that were predicted to target *NBS-LRR*, *STK* or *RLK* in this study. Among them, the total expression abundance of 6 down-regulated phasiRNAs decreased from 4091.77 to 2330.79 RPM, with a decrease of 1760.98 RPM. And they targeted 62 transcripts of *NBS-LRR*, *STK* or *RLK*, with 30 transcripts that increased in expression, 7 that decreased and 25 remained unchanged. The total expression abundance of 6 up-regulated phasiRNAs increased from 316.07 to 590.39 RPM, with an increase of 274.32 RPM. And they targeted 31 transcripts of *NBS-LRR*, *STK* or *RLK*, with 13 that increased in expression, 6 that decreased and 12 remained unchanged. Thus, the results indicated that the down-regulated phasiRNAs had higher abundance and larger variation range, and were more effective. Their expression changes were basically negatively correlated with that of their target genes. And most of these differentially expressed target genes were up-regulated. Specifically, the number of the target resistance genes were most, which targeted by the two down-regulated phasiRNAs 11_1518_166(+) and 11_1518_229(+).

## Discussion

### Response of sRNA to ABA

Exogenous ABA induces tomato sRNA to respond to ABA. As the most in-depth study of sRNA, miRNAs directly or indirectly participate in the regulation of plant growth and development, the response to biotic and abiotic stress, and other life activities by splicing target mRNA and inhibiting its translation or interaction with other sRNAs [56]. Therefore, we carried out an overall normalization analysis of the expression levels of tomato tRFs, phasiRNAs and miRNAs in this study. It was found that the expression of miRNAs was much higher than that of phasiRNAs and tRFs in C1D or A1D, and the tRFs abundance was the least. It is speculated that the physiological regulation of miRNAs is greater than that of phasiRNAs and tRFs. To some extent, the function of miRNAs may even mask that of phasiRNAs and tRFs. Therefore, in exploring the response of tomato tRFs and phasiRNAs to exogenous ABA, it is necessary to normalize the expression of these three sRNAs as a whole. In addition, the abundance of miRNAs, tRFs and phasiRNAs in tomato decreased after spraying exogenous ABA, which indicated that exogenous ABA could inhibit the expression of miRNAs, tRFs and phasiRNAs.

### The features of tRFs

In this study, most of the tRFs originated from tRNA^Ala^, and it tended to produce tRF^Ala^ with length of 20 nt by cutting at tRNA^Ala^ 5^**’**^ terminal guanine site and D loop uracil site. Previous studies have shown that the tRF expression profiles of different species and even different tissues of the same plant will change in varying ranges under stress, but in general, the expression of tRF^Asp^ will increase and the expression of tRF^Glu^ will decrease [10]. Similarly, in this study, the expression of tRF^Asp^ was up-regulated and tRF^Glu^ was down-regulated after treatment of exogenous ABA. It is speculated that the up-regulation of tRF^Asp^ and the down-regulation of tRF^Glu^ are common ways for plants to cope with stresses. The length of tRFs in C1D and A1D was mainly 20 nt, which was similar to the length distribution of tRFs predicted in *Arabidopsis thaliana* [4]. It was indicated that the length of tRFs were not be changed by ABA. However, compared with C1D, the total expression level of tRFs in A1D was significantly reduced, and the expression of tRF^Ala^ with length of 20 nt decreased most, indicating that spraying of exogenous ABA inhibited the expression of tRFs, especially restrained the production of 20 nt tRF^Ala^.

Most of the predicted tRFs fall within the 5e-tRF range, which is similar to the type distribution of tRFs in human prostate cancer cells [33], *Schistosoma japonicum* [11], *Arabidopsis thaliana*, *Oryza sativa* and *Physcomitrella patens* [4]. It indicates that tRF is not cleaved at any position of tRNA, mainly produced at tRNA 5^**’**^terminal and it is speculated that the way of processing tRFs is evolutionarily conserved. In addition, although the expression of 5e-tRFs decreased after exposed to exogenous ABA, 5e-tRFs still played an absolute dominant role in A1D, which was similar to the type distribution of tRFs in leaves, apical meristems, pollens and endosperms of *Brassica rapa* under high temperature stress [10], indicating that stress treatment did not alter the way of tRNA processed to produce tRF. According to classification criteria by Olvedy et.al [44], the fragment between the tRNA start position and the last third base of the D-loop “hairpin” is 5e-tRF. The standard length of 5e-tRF is just 20 nt according to the tRNA “clover” secondary structure, which indicates that most of the tRFs identified by this sequencing start from tRNA 5^**’**^ terminal and end to D-loop. The terminal 5^**’**^ base of 5e-tRF is mostly guanine in C1D and A1D, which is consistent with the base preference in rice pollen and *Arabidopsis thaliana* [35, 37]. It is speculated that it may be related to tRNA 5^**’**^terminal receptor brachial base preference to guanine, which seems to be related to the production and stability of tRFs [35]. The base preference of 5e-tRFs in A1D and C1D was basically the same, indicating that the base preference is conservative and would not be changed by the treatment of exogenous ABA.

Overall, the treatment of exogenous ABA, while inducing or inhibiting the expression of part of tRFs, did not alter the origin, length, type and base preference of tomato tRF.

### Differential expression of tRFs induced by ABA

Overall, the number of down-regulated tRFs (82) was more than that of up-regulated tRFs (74) in the 156 remaining tRFs with abundances more than 2 RPM at least in C1D or A1D. Further analysis of differentially expressed tRFs with abundances more than 10 RPM at least in C1D or A1D, 51 tRFs showing that the number of down-regulated tRFs (41) was more than that of up-regulated tRFs (10). Similarly, the total abundance of down-regulated expression of tRF (from 33,315.52 to 26,572.77 RPM) was much higher than that of up-regulated expression (663.41 to 840.83 RPM), suggesting that the inhibition of tRF expression by ABA was stronger than promotion. Reportedly, plant tRF expression profiles change in varying ranges under biotic and abiotic stresses. In *Arabidopsis thaliana* roots, the abundance of tRFs increased under phosphorus deficiency, drought stress or pathogen infection [25, 37]. However, the abundance of most of tRFs in *Brassica rapa* leaves, embryos, endosperms and ovules decreased under high temperature [10]. Here, most of tRFs in tomato leaves treated with low concentration of ABA were down-regulated in this study. Accordingly, it is speculated that plant can cope with stress by regulating the expression of tRFs, but the specific mechanism is still unclear and needs further study. In addition, the amount of a tRF^Ala^ (ID:ENSRNA049443699:1–20) was much higher than the others, which may play an important role.

### *PHAS* loci

Of the 137 phasiRNAs, 110 originated from protein-coding genes, which is consistent with the conclusion proposed that phasiRNAs in dicotyledons were mainly derived from protein-coding genes [63]. Furthermore, our results presented that most of them were derived from *NBS-LRR* and *RLK* resistance genes, which are similar to the results of other studies [50, 61, 65]. In soybean and *Medicago truncatula*, miR2118 triggered *TIR-NBS-LRR* loci to produce phasiRNAs to resist pathogen infection, and similar target loci were also found in rice and Arabidopsis [26, 55]. Combined with the results of predicting phasiRNAs target genes, it inferred that phasiRNAs may play an important role in plant disease resistance.

### Differential expressions of phasiRNAs induced by ABA

The expression of phasiRNAs altered differentially when treated by exogenous ABA. Here 68 phasiRNAs were detected differentially expressed. Although the number of down-regulated phasiRNAs (29) was lower than that of up-regulated ones (39), the overall abundance and variation range of down-regulated phasiRNAs (from 10,715.98 to 6710.06 RPM) were much higher than that of the up-regulated phasiRNAs (3715.64 to 5529.62 RPM). It indicated that the inhibitory effect of ABA on phasiRNAs expression was stronger than facilitation. Previous studies have shown that the expression of phasiRNAs changed at varying ranges in *Arabidopsis thaliana* under phosphorus deficiency conditions [25], in *Populus trichocarpa* under drought [51] and in tomato infected with *B. cinereal* [61]. In this study, the phasiRNA expressions were inhibited in generally in tomato leaves after exposure to exogenous ABA, presenting that ABA can regulate phasiRNAs expressions and further proved that phasiRNA may be involved in plant responses to different biotic and abiotic stresses.

### phasiRNAs involved in stress responses

#### The phasiRNAs that target TFs

Transcription factors (TFs), also known as trans-acting factors, activate or inhibit the transcriptional expression of target genes by binding to specific DNA sequences called cis-acting element in the promoter region of target genes [48]. A growing number of researches have shown that in addition to plant growth and development, plant TFs play an increasingly important role in plant adaptation to a variety of biotic and abiotic stresses. The expression of many TFs genes including *AP2/ERF*, *MYB*, *bHLH* and *WRKY* can be induced by a variety of biological and abiotic stresses, such as high salt, drought, low temperature, plant hormones, pathogen infection and so on [16, 27, 47, 58]. Over-expression or knockout of TF genes can affect the tolerance of plants to multiple stresses. For example, over-expression of Arabidopsis AP2/ERF gene *RAP2.2* can improve plant resistance to gray mold, but the susceptibility greatly increased after knocking out the gene [67]. And over-expression of WRKY gene *BcWRKY46* and *CaWRKY40* can improve the tolerance of tobacco to low temperature and pepper to heat and bacterial wilt, respectively [16, 57].

Here, 18 transcripts of seven kinds of TFs were predicted to be the targets of 7 phasiRNAs, of which targeted by up-regulated phasiRNAs were either undetected or their expression remained unchanged. However, the TFs targeted by down-regulated phasiRNAs showed that most of the transcripts with enhanced expressions, none had reduced expression, and some remained unchanged or not be detected. Since the abundance and variation of the down-regulated phasiRNAs were higher than that of the up-regulations, thus it was speculated that the effects of the down-regulations were also stronger.

Therefore, the application of exogenous ABA inhibited the expression of phasiRNAs that targeted the TFs and up-regulated TFs expressions, which probably improve plant tolerance to various biotic and abiotic stresses. Moreover, we found that the abundances of TFs targeted by phasiRNAs in both the control and treatment groups were relatively low (FPKM < 15), which is presumed to closely related to their physiological effects. TFs regulate the expression of multiple downstream functional genes by binding to cis-acting elements in the promoter region of target genes, and slight changes in the abundance of TFs will trigger a series of downstream signal amplification cascade reactions, which will cause a series of physiological changes [60].

#### The phasiRNAs that target R genes

Reportedly, there are multiple *PHAS* loci in the R gene protecting the plant against pathogens, and the resulting phasiRNAs also target the R genes [61]. Many phasiRNAs can regulate other *NBS-LRR* loci in a cis- or trans-regulatory manner after targeting a *NBS-LRR* transcript, presenting amplification cascade effect to enhance plant disease resistance [65]. Similarly, most phasiRNAs originated from *NBS-LRR* and *RLK* resistance genes, and most differentially expressed phasiRNAs were predicted to target resistance genes *NBS-LRR*, *STK* and *RLK* in this study. NBS-LRR disease-resistant protein can directly or indirectly recognize pathogens, stimulate downstream signal cascade reaction, and then trigger defense response, hypersensitivity reaction or cell apoptosis [9, 17]. In the present study, three down-regulated phasiRNAs targeted seven NBS-LRR transcripts, of which three were elevated and four were unaltered after treated with exogenous ABA. Three up-regulated phasiRNAs targeted fourteen NBS-LRR transcripts, of which six were elevated, four were lowered and four were unaltered. In all, the target *NBS-LRR* gene expressions were 9 up-regulations, 8 unchanges, and 4 down-regulations. Regarding the relationship of phasiRNAs and their resistance genes detected in this study, we discussed them together later.

As an important member of the R gene family, STK exists widely in various plants, and one of its functions is to participate in the transmission of environmental and pathogen stress signals [12]. Up to date, 57 STK members have been found in *Arabidopsis thaliana*, of which 23 have been confirmed to be involved in the response to salicylic acid, ABA, high salinity, high osmotic, cold and heat stress [45]. Rice bacterial blight resistance gene *Xa21* [53], wheat leaf rust resistance gene *Lr10* [21] and tomato pseudomonas resistance gene *Pto* [40] all contain STK domains. In this study, 4 down-regulated phasiRNAs targeted 17 STK transcripts, with 9 that were increased in expression, 3 that were decreased and 5 remained unchanged. Four up-regulated phasiRNAs targeted 13 STK transcripts, with 6 that were increased in expression, 2 that were decreased and 5 remained unchanged.

RLK belongs to the STK family and is named for its structure and function similar to animal receptor kinase (tyrosine protein kinase), and plays an important role in plant growth and development, signal transduction and various stress responses by sensing external signals [3, 31]. It was found that *Arabidopsis thaliana* RLK family gene *WAKL4* is up-regulated after Cu^2+^, Zn^2+^ and Ni^2+^ treatment, *WAKL4* gene T-DNA insertion mutant has hypersensitivity to Cu^2+^ and Zn^2+^, and the tolerance of *Arabidopsis thaliana* over-expressing *WAKL4* gene to Ni^2+^ was enhanced [23]. Kim and Hwang found a pepper (*Capsicum annuum*) receptor-like cytoplasmic protein kinase gene *CaPIK1* was transcriptionally activated by infection with Xanthomonas campestris pv. vesicatoria and the content of salicylic acid in pepper over-expressing *CaPIK1* gene is higher than that in wild type, and when plant was infected with pathogenic bacteria, CaPIK1 protein regulates defense response through salicylic acid pathway [30]. In this study, five down-regulated phasiRNAs targeted 38 RLK transcripts, of which 18 were elevated, 4 were decreased and 16 were unaltered following ABA treatment. Three up-regulated phasiRNA targeted 4 RLK transcripts, of which one was elevated and 3 were unaltered.

In all, transcripts of R genes targeted by down-regulated phasiRNARs were mostly increased or unchanged in expression level (30 were elevated, 7 were decreased, and 25 were unaltered), exhibiting the down-regulated phasiRNARs were negative with their target R genes. But the correlation between the up-regulated phasiRNAs and their targets was not obvious (13 were elevated, 6 were decreased, and 12 were unaltered). It was speculated that the effects of the down-regulations were stronger, for the same reason as above, the abundance and variation of the down-regulated phasiRNAs (from 4091.77 to 2330.79 RPM) were much higher than that of the up-regulations (316.07 to 590.39 RPM). It may also be the result of the interaction of other non-coding RNAs.

Importantly, the three phasiRNAs of 6_1497_500(−) (from 1628.21 to 1188.46 RPM), 11_1518_166(+) (1368.32 to 291.07) and 11_1518_229(+) (72.79 to 37.35), targeted multiple genes (including TFs and R genes), and their abundance varied with a larger extent by ABA. The results exhibited that they probably exert key roles in plant stress adaptions. Their functions should be for further investigation.

Overall, ABA caused down-regulation of phasiRNAs, while TFs and resistance protein genes were up-regulated, showing a negative correlation between the phasiRNAs and the target genes. It presented that ABA may improve the environmental and disease resistance of plants, and the results complied with our field results (data not shown).

## Conclusions

In conclusion, the application of exogenous ABA inhibited the expressions of tRFs and phasiRNAs in tomato, significantly reduced the abundance of phsaiRNAs related to stress resistances, and up-regulated the expressions of corresponding target genes associated to stress and disease resistance, including TFs of AP2/ERF, MYB, NAC, ARF, GRAS, bHLH, WRKY and R genes of *NBS-LRR*, *STK*, *RLK*. Combining the previous analyses of comparative transcriptome and miRNAs responding to ABA, it is presented that ABA can improve plant adaptability to various biological and abiotic stresses by regulating the expression and interaction of sRNAs such as miRNAs, phasiRNAs and tRFs with their target genes.

## Supplementary information

**Additional file 1 Table S1**. List of tRFs identified in this study and their expression levels. **Table S2**. The differential expression levels in the control (C1D) and ABA-treatment (A1D) libraries of tRFs after reducing noise. **Table S3**. List of differentially expressed tRFs with a minimum 10 RPM value in one library. **Table S4**. List of phasiRNAs identified in this study and their expression levels. **Table S5**. The differential expression levels in the control (C1D) and ABA-treatment (A1D) libraries of phasiRNAs. **Table S6**. List of differentially expressed phasiRNAs with a minimum 10 RPM value in one library. **Table S7**. The target genes of phasiRNAs. **Table S8**. The expression patterns of phasiRNAs targets

## Data Availability

All of the sequencing reads were deposited in Sequence Read Archive database under the Accession number of PRJNA209403 (https://trace.ncbi.nlm.nih.gov/Traces/sra_sub/sub.cgi?subid=1943955 &from = list&action = show:submission).

## Abbreviations

A1D: Treatment group
ABA: Abscisic acid
C1D: Control group
NBS-LRR: Nucleotide binding site-Leucine rich repeat
phasiRNA: Phased, secondary, small interfering RNA
RLK: Receptor-like kinase
RPM: Reads per million
STK: Serine/threonine-protein kinase
TF: Transcription factor
tRF: tRNA-derived fragment
bHLH: Basic helix-loop-helix
